# Specific gut microbiome signatures and the associated pro-inflamatory functions are linked to pediatric allergy and acquisition of immune tolerance

**DOI:** 10.1038/s41467-021-26266-z

**Published:** 2021-10-13

**Authors:** Francesca De Filippis, Lorella Paparo, Rita Nocerino, Giusy Della Gatta, Laura Carucci, Roberto Russo, Edoardo Pasolli, Danilo Ercolini, Roberto Berni Canani

**Affiliations:** 1 Department of Agricultural Sciences, University of Naples Federico II, Naples, Italy; 2 Task Force on Microbiome Studies, University of Naples Federico II, Naples, Italy; 3 Department of Translational Medical Science, University of Naples Federico II, Naples, Italy; 4 ImmunoNutritionLab at CEINGE-Biotecnologie Avanzate s.c.ar.l, University of Naples Federico II, Naples, Italy; 5 Department of Pharmacy, University of Naples Federico II, Naples, Italy; 6 European Laboratory for the Investigation of Food-Induced Diseases, University of Naples Federico II, Naples, Italy

**Keywords:** Clinical microbiology, Microbial ecology

## Abstract

Understanding the functional potential of the gut microbiome is of primary importance for the design of innovative strategies for allergy treatment and prevention. Here we report the gut microbiome features of 90 children affected by food (FA) or respiratory (RA) allergies and 30 age-matched, healthy controls (CT). We identify specific microbial signatures in the gut microbiome of allergic children, such as higher abundance of *Ruminococcus gnavus* and *Faecalibacterium prausnitzii*, and a depletion of *Bifidobacterium longum*, *Bacteroides dorei*, *B. vulgatu*s and fiber-degrading taxa. The metagenome of allergic children shows a pro-inflammatory potential, with an enrichment of genes involved in the production of bacterial lipo-polysaccharides and urease. We demonstrate that specific gut microbiome signatures at baseline can be predictable of immune tolerance acquisition. Finally, a strain-level selection occurring in the gut microbiome of allergic subjects is identified. *R. gnavus* strains enriched in FA and RA showed lower ability to degrade fiber, and genes involved in the production of a pro-inflammatory polysaccharide. We demonstrate that a gut microbiome dysbiosis occurs in allergic children, with *R. gnavus* emerging as a main player in pediatric allergy. These findings may open new strategies in the development of innovative preventive and therapeutic approaches. Trial: NCT04750980.

## Abstract (web summary)

Here, the authors profile the taxonomic composition and genetic potential of the gut microbiome of children with food or respiratory allergies and find that the gut metagenome of these patients is characterized by higher proinflammatory potential and reduced capacity of degrading complex polysaccharides, with *Ruminococcus gnavus* playing a central role.

## Introduction

Prevalence of allergies among children has become an increasing problem in the last few decades^1^. Although genetic predisposition could be relevant for allergy development, several environmental factors have been also suggested. Many of these environmental factors, such as antibiotic use, caesarean delivery, and dietary habits, act mainly modulating the gut microbiome^2^. Therefore, it is not surprising that increasing evidence indicated potential links between the gut microbiome and the development of allergy. Although differences in the microbial signatures identified, the research highlighted the presence of gut microbiome dysbiosis in food and respiratory allergies^3–7^. Dysbiosis refers to an unbalance in the microbiota composition and function such that it breaks gut homeostasis and contributes to diseases^8^. Gut microbiome dysbiosis may affect the integrity of the intestinal epithelial barrier, leading to the entry of antigens in the bloodstream and the abnormal stimulation of the immune system^9^, which in part could explain the relationships even with respiratory allergies^10^. Indeed, microbial colonization of the gut mucosal surfaces plays a pivotal role in the maturation of the host’s immune system^11^. Microbial metabolites, in particular short-chain fatty acids (SCFAs) produced from microbial fermentation of undigested fiber, may play a role in the maintenance of epithelial integrity and in the stimulation of immune tolerance^12,13^. Accordingly, several studies reported low fecal levels of SCFAs in allergic subjects^7,14,15^, while children with a microbiome depleted of genes related to fiber fermentation showed a higher probability to develop allergic sensitization^16^.

The gut microbiome composition in early life has been also proposed as predictive for food allergy resolution. Indeed, children with cow’s milk allergy (CMA) showing higher levels of Clostridia in the first months of life had a higher probability to acquire immune tolerance at the age of 8 years^17^.

However, although several studies reported a link between gut microbiome and allergy, a causative role is largely undefined. The study of mechanistic causal relationships between gut microbiome, host immune system and allergy development is gaining insights by the use of germ-free animal models. Mice colonized with Clostridia seem to have an improved gut permeability, that protects against allergen sensitization^18^, while we have recently demonstrated that mice colonized with the gut microbiome from healthy infants were protected against the anaphylactic responses to the cow’s milk allergen β-lactoglobulin^19^.

Manipulation of the gut microbiome could be a promising approach for novel preventive and therapeutic strategies against allergy. Therefore, identifying microbial signatures typical of allergic diseases and understanding the functional potential of the disrupted microbiome is of primary importance for the design of such innovative strategies.

The MATFA (Microbiome As potential Target for innovative preventive and therapeutic strategies for Food Allergy) project was designed to comparatively evaluate the gut microbiome features of children affected by food (FA) and respiratory (RA) allergies. We evaluated the gut microbiome of allergic children, exploring their taxonomic composition, as well as the genetic potential. The gut metagenome of allergy was characterized by higher pro-inflammatory potential and a reduced capacity of degrading complex polysaccharides, where *R. gnavus* seems to play a central role. In addition, the occurrence of a strain-level selection in the gut microbiome of allergic children was also identified, with specific strains of *R. gnavus* showing higher prevalence in allergic subjects. Finally, we also demonstrated that specific gut microbiome signatures at baseline can be predictable of immune tolerance acquisition, suggesting a possible influence of the microbiome on the natural history of FA.

## Results

### Study subjects

From January 1st 2017 to June 30th 2020, 90 subjects with a sure diagnosis of (immunoglobulin) IgE-mediated allergy and 30 age-matched healthy controls (CT) were evaluated for the study. All subjects accepted to participate and stool samples were collected from each child at the diagnosis. Six stool samples failed in sequencing procedures, then shotgun metagenomics analysis was performed on 114 subjects: 30 with respiratory allergies (RA) (15 with allergic asthma and 15 with oculorhinitis), 55 with FA, ad 29 CT.

Among RA patients, 11 were allergic to one allergen (house dust mites), while the other 19 resulted allergic to ≥2 allergens (pollens, house dust mites and dog epithelia). Among FA children, 22 resulted allergic to one allergen (11 cow’s milk; 6 hen’s egg; 3 nuts; 2 peach), while the other 33 resulted allergic to ≥ 2 food allergens (18 cow’s milk and hen’s egg; 2 cow’s milk and food other than hen’s egg; 8 hen’s egg and food other than cow’s milk; 5 food allergens different from cow’s milk and hen’s egg), 30 presented gastrointestinal symptoms (20 vomiting; 16 diarrhea), 30 cutaneous symptoms (urticaria). Baseline main demographic and clinical characteristics of the study population were reported in Table 1.

**Table 1:** Main demographic and clinical characteristics of the study population.

	Patients with respiratory allergy	Patients with food allergy	Healthy controls	*p* respiratory allergy *vs* food allergy	*p* respiratory allergy vs healthy controls	*p* food allergy vs healthy controls
N.	30	55	29	–	–	–
Male, *n* (%)^a^	21 (70)	34 (61.8)	15 (51.7)	ns	ns	ns
Spontaneous delivery, *n* (%)^a^	16 (53.3)	32 (58.2)	16 (55.2)	ns	ns	ns
Born at term, *n* (%)^a^	30 (100)	55 (100)	29 (100)	–	–	–
Birth weight, gr (mean, SD)^b^	3207 (372.8)	3241.8 (453.4)	3145.9 (546.3)	ns	ns	ns
Age at diagnosis, months (mean, SD)^b^	57.8 (10.9)	14 (15.1)	–	<0.001	–	–
Age at enrollment, months (mean, SD)^b^	57.8 (10.9)	57.4 (11)	62.1 (10.1)	ns	ns	ns
Breastfeeding for at least 4 weeks, *n* (%)^a^	15 (50)	41 (74.5)	20 (69)	0.023	ns	ns
Duration of breastfeeding, months (mean, SD)^b^	7.4 (6.2)	8.6 (7.4)	9.5 (9)	ns	ns	ns
Weaning age, months (mean, SD)^b^	5.3 (0.8)	5.3 (1.2)	5.1 (0.7)	ns	ns	ns
Familial allergy risk, *n* (%)^a^	19 (63.3)	40 (72.7)	0 (0)	ns	<0.001	<0.001

*ns* not significant, *SD* standard deviation.

^a^The χ2 test was used as statistical test.

^b^Two-tailed Student’s *t* test was used as a statistical test.

All study subjects were followed at the Center for at least 36 months after the enrollment. In children with FA the possible acquisition of immune tolerance was assessed every 12 months by the results of skin prick tests, serum specific IgE levels and oral food challenge. Similarly, healthy controls were followed by the physicians involved in the study for the possible occurrence of any allergic conditions for 36 months.

At the end of 36-month follow-up period, 17 out of 55 (30.9%) children with FA acquired immune tolerance (4 at 12 months, 8 at 24 months, 5 at 36 months). All healthy controls remained free from any allergic disease during the 36 months follow-up.

### Specific microbial signatures are associated with the allergy state

**Fig. 1:**
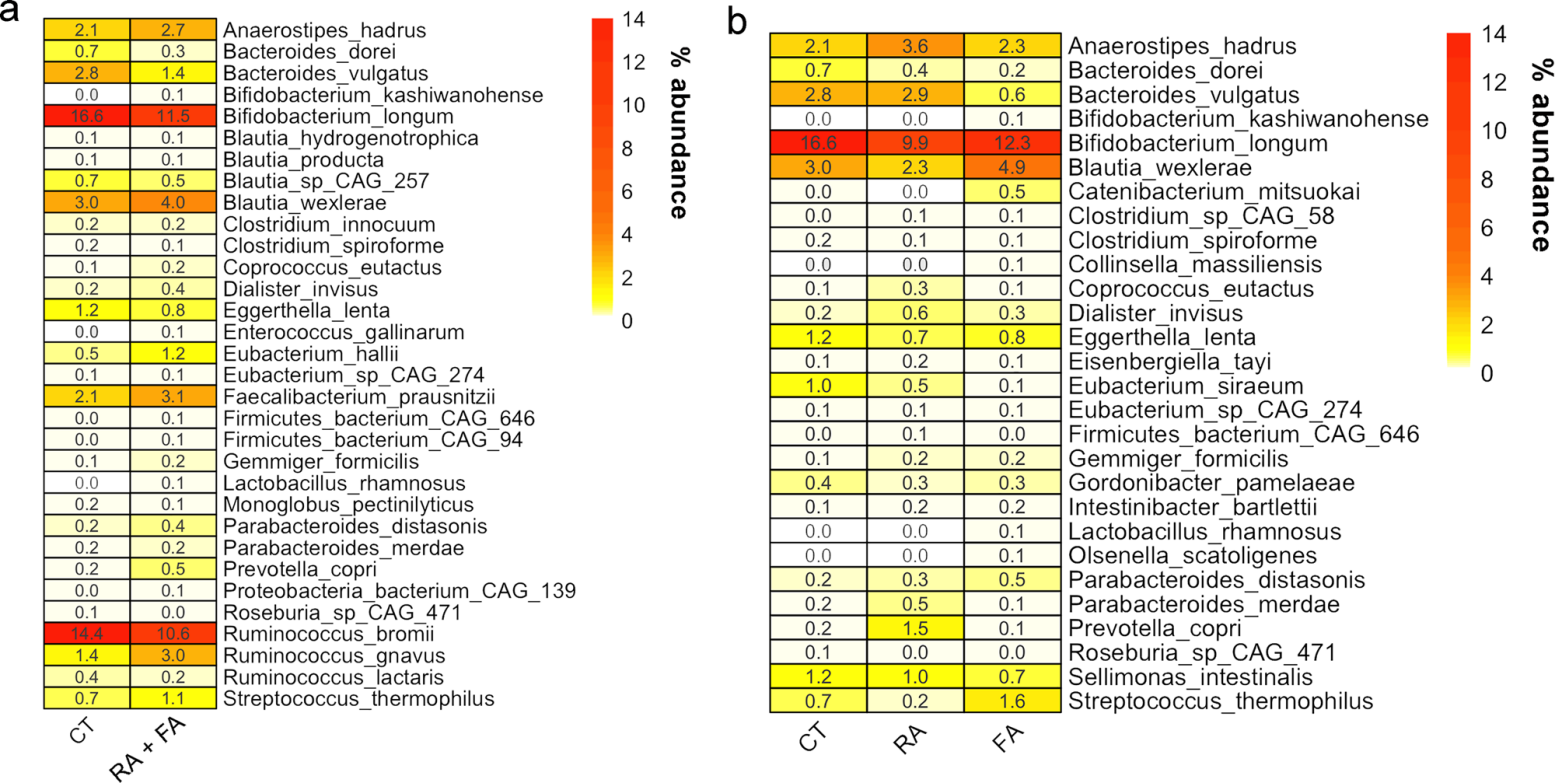
Microbial signatures in allergic children gut microbiome. Heatplot reporting the average relative abundance (%) of microbial taxa significantly different between healthy (CT) and allergic children (**a**) or healthy children (CT) and children with food (FA) or respiratory (RA) allergies (**b**), as defined by Wilcoxon test (FDR *q* < 0.1).

We did not find significant differences in the overall gut microbiome taxonomic composition according to the disease status (CT vs RA + FA or CT vs FA or RA) by PERMANOVA (Permutational Multivariate Analysis of Variance) computed on Jaccard distance matrix (*p* > 0.05). Therefore, no specific clustering of the subjects was observed in PCoA plots based on Jaccard distance matrix (Fig. S1A, B). In addition, microbial diversity indices were not different in FA compared with CT, while higher diversity was observed in RA (Fig. S2). Firstly, we evaluated the hypothesis that the allergic state (FA or RA) could be associated with specific signatures in the gut microbiome (Fig. 1a). Allergic children showed significantly higher abundance of *R. gnavus*, *Faecalibacterium prausnitzii*, *Dialister invisus*, *Anaerostipes hadrus*, several *Blautia* and *Parabacteroides* species compared with healthy controls (Wilcoxon test, *p* < 0.05). On the contrary, their gut microbiome was depleted of *Bif. longum*, *Bacteroides dorei*, *B. vulgatus* and some fiber-degrading taxa (e.g., *Roseburia* CAG_471, *R. bromii;* Fig. 1a). However, when evaluating the differences associated with the type of allergy, we also identified some allergy-specific signatures. Children with FA showed a microbial pattern characterized by decreased abundance of *B. vulgatus*, and higher levels of *Blautia wexlerae* compared with RA (*p* < 0.05; Fig. 1b). In contrast, *Anaerostipes hadrus* and *Prevotella copri* were higher in subjects with RA compared with both CT and FA patients (Fig. 1b). Since we recently demonstrated the presence of at least 12 different species within *Faecalibacterium* genus, we used the same pipeline^20^ to test the occurrence of *F. prausnitzii* clades in the samples. Interestingly, *F. prausnitzii* clade A (that includes, among others, the strain L2-6, previously linked with atopic dermatitis^21^) was enriched in FA, compared with both RA and CT (chi-squared test, *p* < 0.05).

**Fig. 2:**
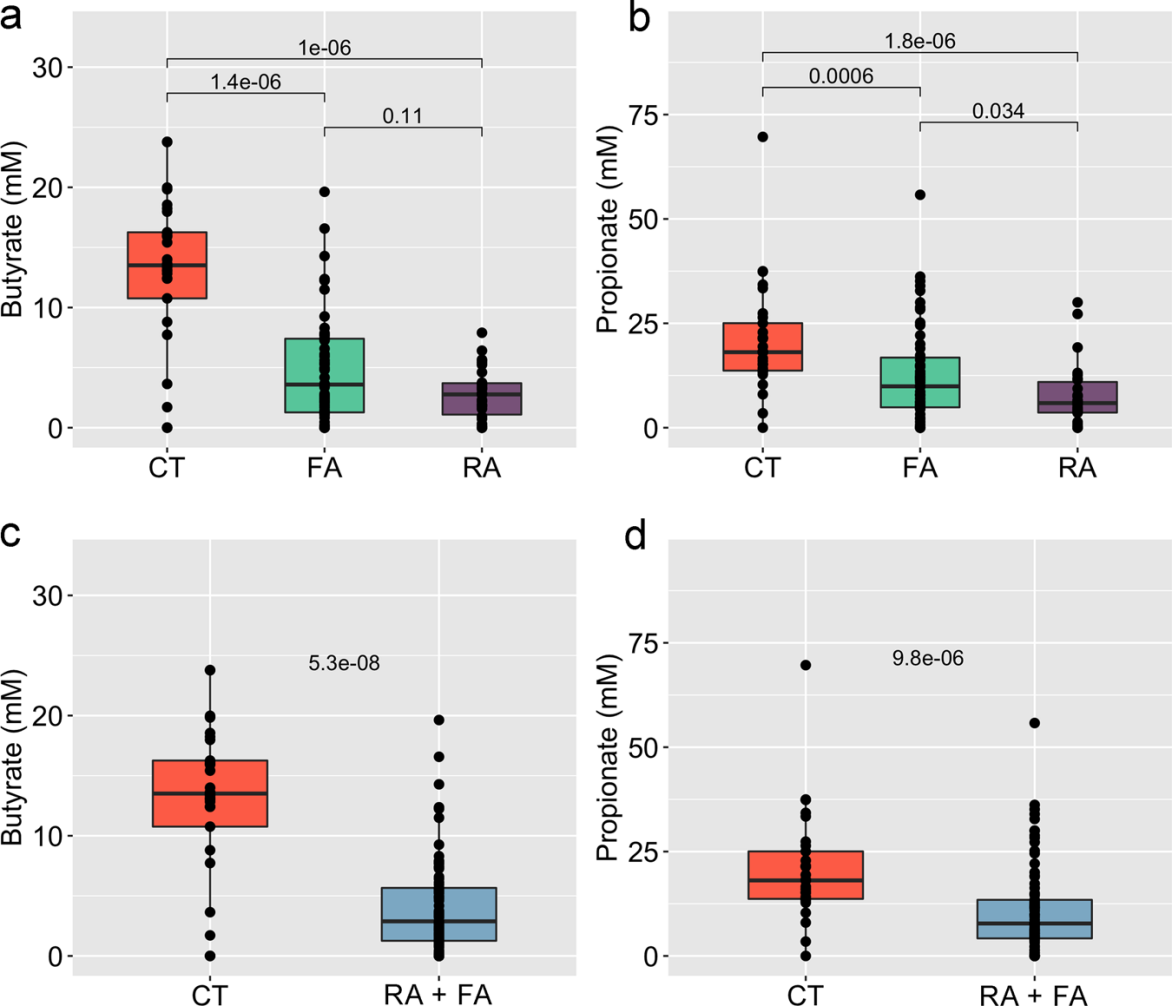
Short-chain fatty acids are depleted in the gut of allergic children. Box plots showing the concentration of butyrate (**a**, **c**) and propionate (**b**, **d**) in faecal samples of allergic and healthy children. Allergic children are included in a unique group (**c**, **d**) or separated according to the type of allergy (**a**, **b**). The significance was tested by applying pairwise Wilcoxon test. Data are obtained from *n* = 29, 55 and 30 biologically independent samples for CT (healthy controls), FA (food allergy) and RA (respiratory allergy), respectively. Boxes represent the interquartile range (IQR) between the first and third quartiles, and the line inside represents the median (2nd quartile). Whiskers denote the lowest and the highest values within 1.5 x IQR from the first and third quartiles, respectively.

We also evaluated differences in the gut microbiome in FA and RA children with sensitization to multiple allergens compared with those having sensitization to a single allergen, but we did not find differences comparing the two groups. Interestingly, fecal levels of butyrate and propionate were consistently higher in CT compared with both allergic groups (Fig. 2).

We used a machine-learning-based classification approach to evaluate if the gut microbiome composition at species-level could discriminate among different conditions. We observed a moderate (area under the curve AUC = 0.64, 95% Confidence Interval, CI: 0.58–0.70) but significant (*p* < 0.01 by computing the statistical test against the null hypothesis of equal AUC for classification of true and shuffled labels) discrimination between healthy and allergic children irrespective of the allergy type. Moreover, we found a high discrimination (AUC = 0.79, 95% CI: 0.72–0.86) when comparing FA and RA, supporting the finding that different microbial taxa are associated with different allergy types.

**Fig. 3:**
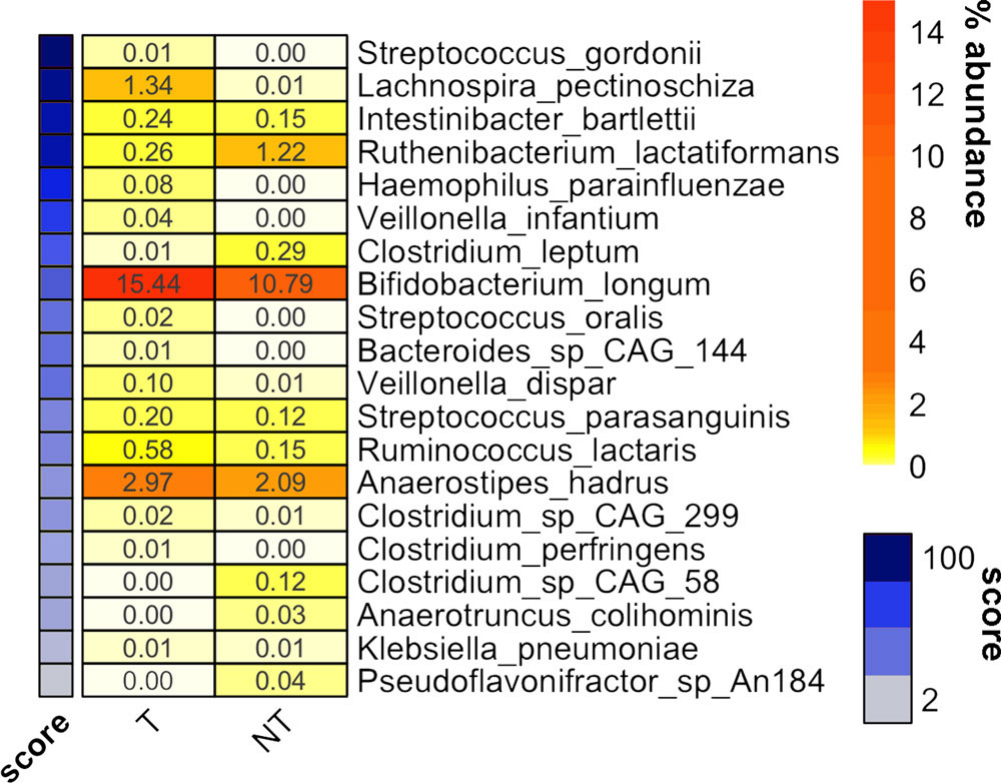
Gut microbiome features may predict the development of immune tolerance. Heatplot reporting the average relative abundance (%) of microbial taxa significantly different between food allergy children developing (T) or not (NT) immune tolerance upon 36 months of exclusion diet, as defined by Wilcoxon test (FDR *q* < 0.1). Taxa are ordered according to the feature importance score from Random Forest classification model, indicated in the side colored bar.

In addition, we found that specific gut microbiome features at baseline (FDR *q* < 0.1) were associated with the acquisition of immune tolerance, suggesting a possible influence of the microbiome on FA disease course (Fig. 3). Children with CMA who developed immune tolerance (T) showed higher abundance of *Bif. longum, Lachnospira pectinoschiza* and *A. hadrus* at diagnosis, as well as lower levels of *Ruthenibacterium lactatiformans* and *Clostridium leptum* if compared with children who did not acquire immune tolerance (NT), while the baseline fecal level of butyrate and propionate were similar into the two groups. Consistently, the machine-learning-based classification showed a good discrimination in terms of immune tolerance acquisition, with AUC = 0.74 (95% CI: 0.68–0.80) when discriminating between T and NT (Fig. 3), a value that was not affected by the addition of SCFAs concentration to the model.

**Fig. 4:**
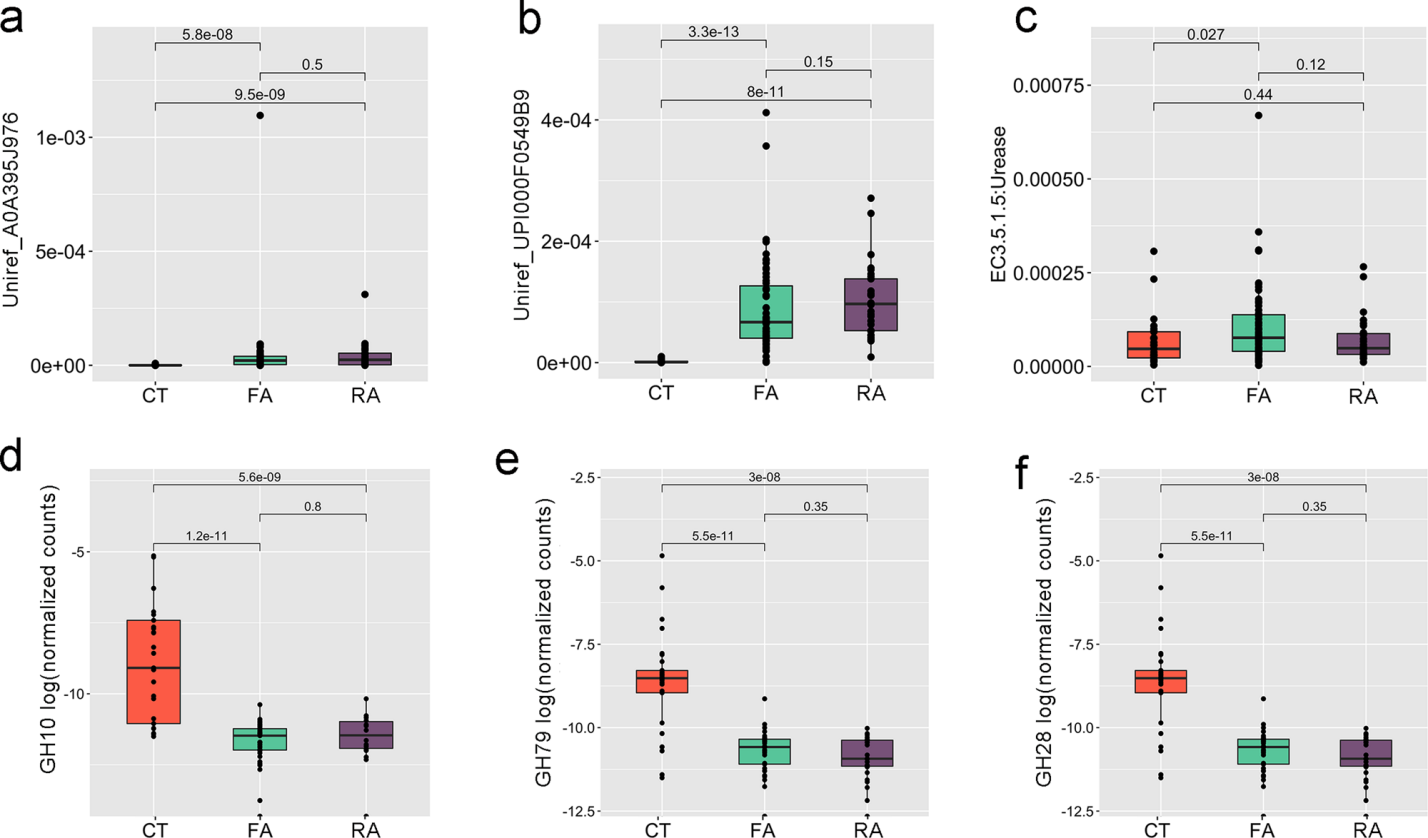
Gut microbiome of allergic children shows a higher inflammatory potential and a reduced ability to degrade complex polysaccharides. Box plots showing the relative abundance of microbial genes involved in lipopolysaccharide biosynthesis (**a**, **b**), urea (**c**) and fiber degradation (**d**–**f**) in healthy (CT), food (FA) and respiratory (RA) allergic children. Data of (**a**–**c**) are reported as relative abundance (number of reads/total number of reads per sample) from HUMAnN3 analysis; data of (**d**–**f**) are reported as normalized counts [log_10_ (number of CAZy hits/total number of genes per sample)]. UniRef_ A0A395J976: O-antigen/teichoic acid export membrane protein; UniRef_UPI000F05499B: LPS biosynthesis protein. Boxes represent the interquartile range (IQR) between the first and third quartiles, and the line inside represents the median (2nd quartile). Whiskers denote the lowest and the highest values within 1.5 x IQR from the first and third quartiles, respectively. The significance was tested by applying pairwise Wilcoxon test. Data are obtained from *n* = 29, 55, and 30 biologically independent samples for CT, FA and RA, respectively.

We used HUMAnN3 to define the functional potential of the gut metagenome and found that the gut microbiome of allergic children was characterized by higher inflammatory potential. Indeed, genes involved in the biosynthesis of the bacterial lipopolysaccharide (LPS; UniRef_A0A395J976 and UPI000F05499B) were more abundant in FA and RA compared with CT (*p* < 0.05, Fig. 4). Moreover, genes coding for urease (E.C. 3.5.1.5) were also enriched in allergic children, showing higher microbial potential for urea degradation with consequent ammonia production (*p* < 0.05, Fig. 4). We further focused on the potential of the gut microbiome to degrade complex polysaccharides by evaluating the number of microbial genes aligning to the CAZy database. CAZy Glycoside Hydrolase (GH) families GH_10 (including xylanases and glucanases), GH_79 (including glucuronidases) and GH_28 (including galacturonases) were all depleted in allergic children compared with CT (Fig. 4; *p* < 0.05), showing a decreased potential for fiber degradation. Consistently with the taxonomic results, the taxa contributing to these gene families were *Roseburia* spp. (GH_79), *Bacteroides* spp. (GH_10), *Bacteroides* spp. and *Roseburia* spp. (GH_28), all taxa enriched in CT vs allergic children.

### Allergic children harbor different functional types of *R. gnavus* and *B. longum*

**Fig. 5:**
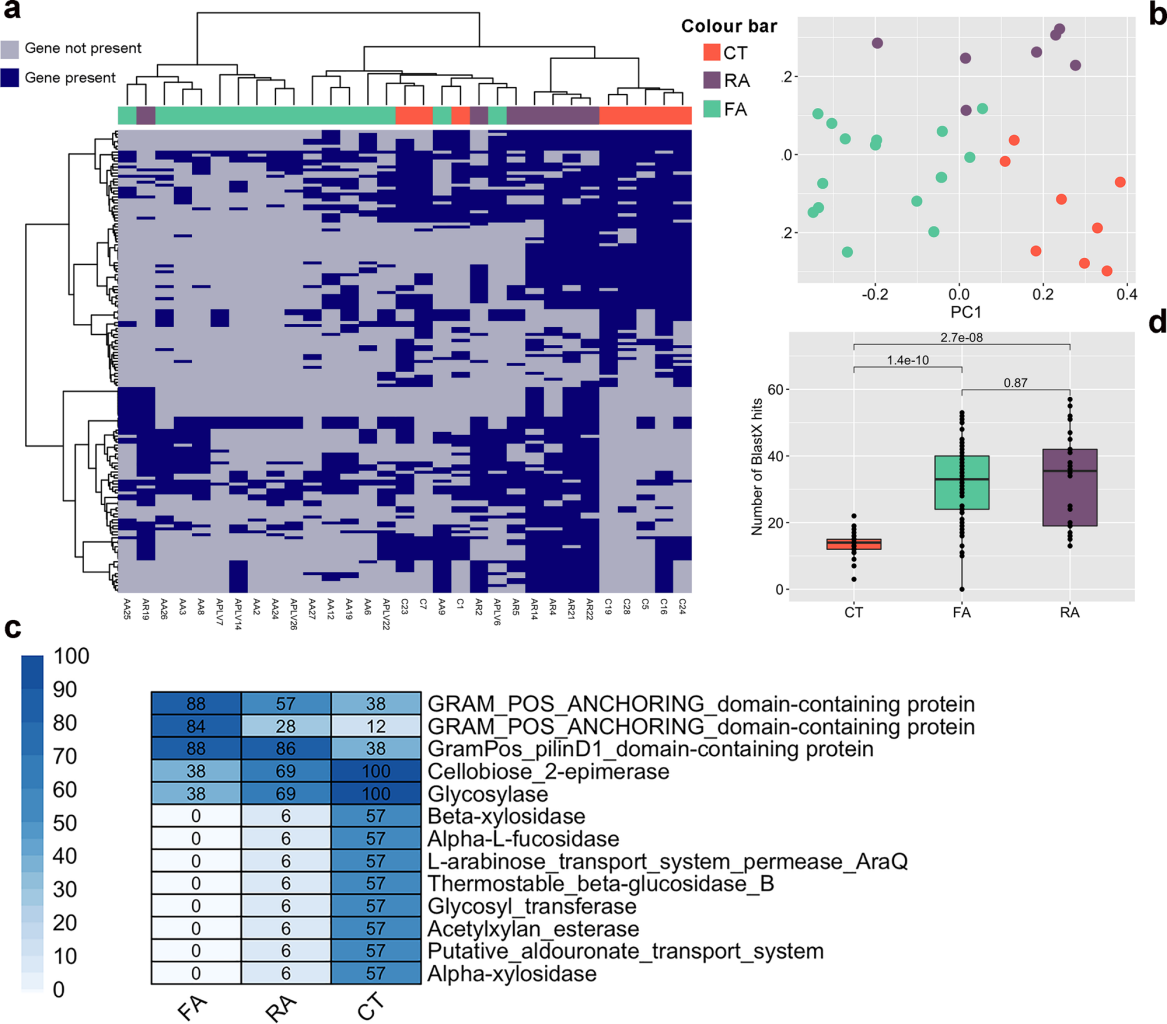
Allergic children harbor a different *R. gnavus* pangenome. **a** Presence and absence of 155 *R. gnavus* genes significantly different between healthy (CT), food (FA) and respiratory (RA) allergic children (blue, present; gray, absent). The significance was tested by applying paired chi-squared tests. **b** Principal coordinates analysis based on presence/absence of the 155 significant genes. **c** Heatplot showing the prevalence (%) of selected significant genes in the three children groups (CT, healthy controls; FA, food allergy; RA, respiratory allergy). The complete list of the 155 significant genes and their prevalence is reported in Supplementary Data 2. **d** Box plots showing the number of hits (> 90% identity over 50% of query length) against *R. gnavus* genes involved in the production of a pro-inflammatory polysaccharide. Boxes represent the interquartile range (IQR) between the first and third quartiles, and the line inside represents the median (2nd quartile). Whiskers denote the lowest and the highest values within 1.5 x IQR from the first and third quartiles, respectively. The significance was tested by applying pairwise Wilcoxon test. Data are obtained from *n* = 29, 55, and 30 biologically independent samples for CT (healthy controls), FA (food allergy) and RA (respiratory allergy), respectively.

We explored the possibility that a selection at strain level occurs in the gut microbiome of allergic children. Firstly, we used a mapping-based approach to define the pangenome of the 10 most abundant species. We carried out this analysis on 11 species (*Bif. bifidum, Bif. breve, Bif. adolescentis*, *Bif. longum*, *B. vulgatus, B. fragilis, B. uniformis, Eubacterium rectale, Akkermansia muciniphila, R. gnavus, R. bromii*) that were selected as the most abundant and present at >2% abundance in at least 80% of the subjects. Among the taxa investigated, we identified differences associated with the allergic state in the pangenome of *Bif. bifidum* and *R. gnavus*. In particular, 76 and 155 pangenes of *Bif. bifidum* and *R. gnavus* respectively, occurred differently in CT and allergic children (either FA or RA; Supplementary Data 2). *Bif. bifidum* pangenome discriminates healthy from allergic children, regardless the type of allergy (FA or RA; Fig. S3A, B). In contrast, when considering *R. gnavus* pangenome, we observed that CT clustered apart from allergic children, who also separated according to the type of allergy (Fig. 5a, b), suggesting the presence of different *R. gnavus* strains. Among *R. gnavus* genes that were enriched in healthy children, we identified several genes involved in complex polysaccharides degradation (e.g., acetylxylan esterase, alpha-L-fucosidase, beta-xylosidase; Fig. 5c). Conversely, allergy-associated strains were characterized by higher potential to adhere to the gut epithelium, having a higher prevalence of genes related to pilin and anchoring factors (Fig. 5c and Supplementary Data 2). We further explored the role of *R. gnavus* specifically looking for the presence of 23 genes related to the biosynthesis of a pro-inflammatory polysaccharide^22^ and we identified a significantly higher number of hits in FA and RA compared with healthy children (Fig. 5d), highlighting the presence of a potential mechanism leading to inflammation in allergic children. We then built a binary matrix showing the presence/absence of the 23 *R. gnavus* genes in the samples and used it in the machine-learning-based classification. We observed a good (AUC = 0.83, 95% CI: 0.77–0.89) and significant (*p* < 0.01) discrimination between healthy and allergic children irrespective of the allergy type. Moreover, we found a discrete discrimination (AUC = 0.72, 95% CI: 0.67–0.77) when comparing children allergic to single or multiple allergens. No discrimination (AUC = 0.50) was found between T and NT groups.

**Fig. 6:**
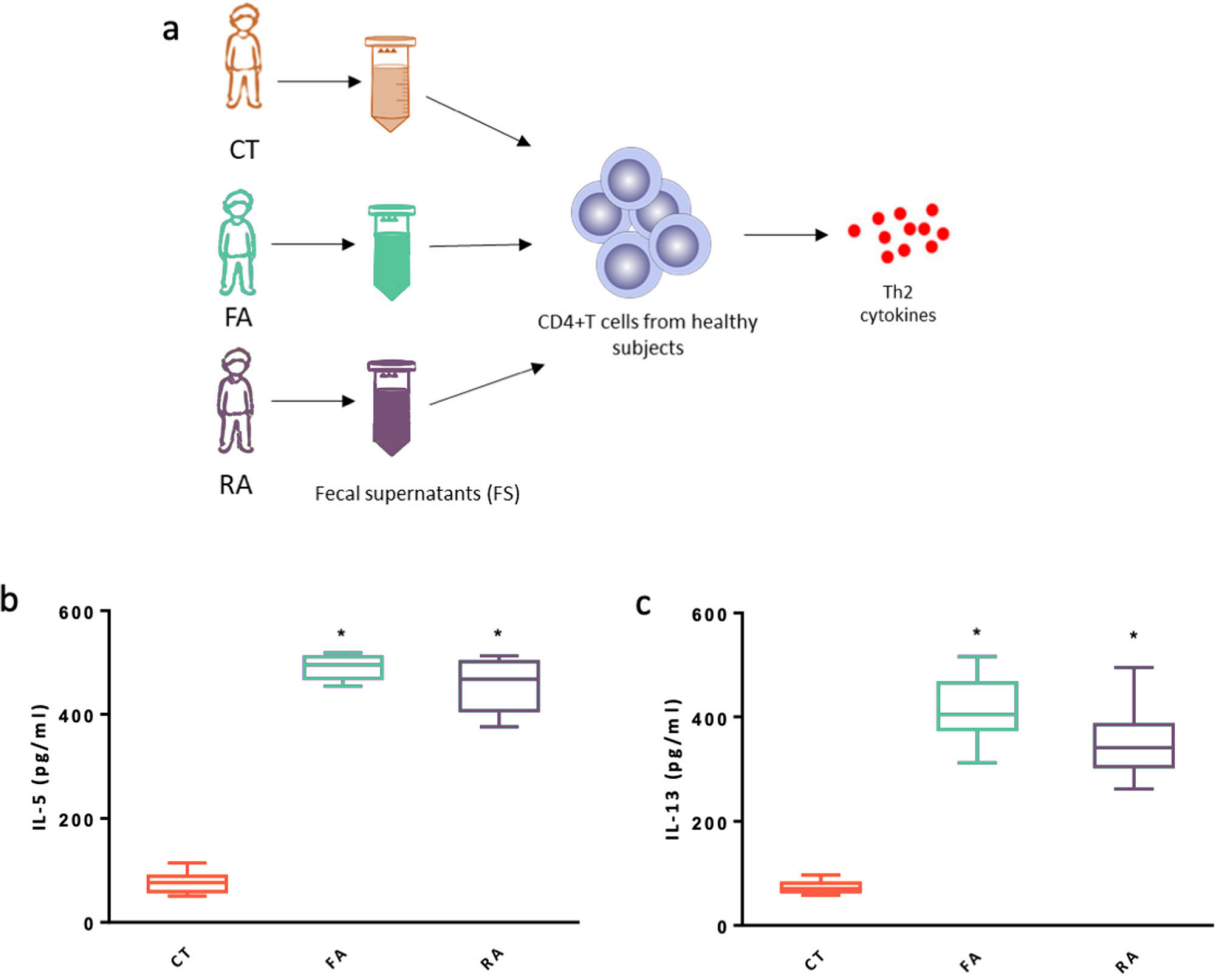
Fecal supernatants from FA and RA children elicit a pro-allergic Th2 cytokines response in human CD4+ T cells. As depicted in the figure, peripheral blood CD4+ T cells from healthy children (*n* = 3) were exposed to fecal supernatants obtained from fecal samples collected from CT, FA and RA subjects (*n* = 3/group) (**a**). Stimulation for 24 h with 100 µl (optimal dose) of fecal supernantants obtained from FA and RA patients, but not with fecal supernatants from CT, induced a significant increase in IL-5 (**b**) and IL-13 (**c**) production by CD4+ T cells. Each fecal supernatant was tested in triplicate on three different peripheral blood CD4+ T cells samples. Data are expressed as mean ± standard deviation. *p = 0.0001, as defined by paired t-test comparing CT vs FA and CT vs RA. (**a**) was generated using GraphPad Prism v. 7.0.

To assess the potential functional effect of the gut microbiome in eliciting an allergic response, we obtained fecal supernatant from CT, FA and RA subjects and tested their effect on peripheral blood CD4+ T cells from healthy children. Fecal supernatants obtained from CT, FA and RA subjects contained very low concentrations of IL-5 and IL-13 (<30 pg/ml). Stimulation with fecal supernantants obtained from FA and RA patients, but not with fecal supernatants from CT, induced a significant increase in IL-5 and IL-13 production by CD4+ T cells (Fig. 6).

### MAGs reconstruction highlights influence of the delivery mode on sub-species diversity

To further explore the effect of allergy on sub-species diversity of the gut microbiome, we also reconstructed Metagenome Assembled Genomes (MAGs). We binned a total of 3357 MAGs from the 117 samples that were clustered into 470 SGBs (Species-level Genome Bins) and taxonomically assigned as reported in Supplementary Data 3. We further analyzed newly reconstructed MAGs to explore the influence of other metadata (i.e., delivery mode and breastfeeding) on sub-species diversity. Interestingly, we identified specific strain-level signatures associated with vaginal or C-section delivery in two species (*Bl. wexlerae* and *B. vulgatus*). For both, we could identify two putative sub-species based on phylogenetic diversity (Fig. S4A, B). In the case of *Bl. wexlerae*, one sub-species was almost exclusively (11 out of 12 MAGs) found in C-section delivered children (Fig. S4A). For *B. vulgatus*, we identified one sub-species found both in C-section and vaginal delivered children, while another was exclusive of vaginal delivery (Fig. S4B). In both cases, no association with allergy was found.

## Discussion

Gut dysbiosis refers to an unbalance in the composition and activity of the gut microbiome. Dysbiosis was previously associated with different conditions, although a cause-effect mechanism remained largely undefined^23^. The link between gut microbiome and allergic diseases was explored in several studies and microbial signatures specific to the different allergies were identified, although a general agreement does not exist^24^. Our experience represents the first use of a shotgun metagenomics approach to look at gut microbiome composition and functional potential in children with IgE-mediated allergy. We identified common features in the gut microbiome of allergic children, regardless the type of allergy (food or respiratory). Indeed, we found an increase in Firmicutes and a decrease in Bacteroidetes taxa in allergic children, as previously reported^24^. In agreement with previous findings, the gut microbiome in FA and RA was characterized by higher abundance of *F. prausnitzii, R. gnavus, Bl. wexlerae, A. hadrus*, as well as lower levels of *Bif. longum, B. dorei, B. vulgatus, R. bromii* and of several other fiber-degrading species compared with healthy controls^16,19,25–28^. *Bif. longum* has been identified as the main *taxon* able to metabolize the human milk oligosaccharides, leading to an increased production of tolerogenic SCFAs^29,30^. In addition, we identified the presence of different *F. prausnitzii* clades (as defined by De Filippis and colleagues^20^). *F. prausnitzii* clade A, previously associated with the Westernized lifestyle^20^, was enriched in FA compared with both RA and CT. Therefore, the increased abundance of *F. prausnitzii* reported in allergic subjects in this and previous studies is probably linked to an increase in this specific clade. Interestingly, Song and colleagues^21^ found an increase in *F. prausnitzii* strain L2-6 (belonging to clade A, according to ref. ^20^) in atopic dermatitis, suggesting a role of this *F. prausnitzii* clade in allergy development.

As previously reported^14^, we did not find differences in gut microbiome taxonomic composition comparing children with single FA or RA vs children sensible to multiple allergens. However, children with multiple allergies showed higher number of *R. gnavus* genes involved in the production of a pro-inflammatory polysaccharide, highlighting the possible presence of different strains of this species. Nevertheless, comparison with previous data is difficult, since existing studies are all based on lower resolution techniques (e.g., 16S rRNA sequencing) and often achieved taxonomic identification at genus level or even above.

In this study, we also  highlighted that the gut microbiome structure in allergy is also reflected in an altered functional potential. The gut microbiome of allergic children showed higher levels of ureases and genes related to LPS biosynthesis. LPS stimulates the production of pro-inflammatory cytokines, thus activating the inflammatory cascade^31^ and it was previously associated with the onset of allergic rhinosinusitis^32^. Ureases are involved in urea degradation and ammonia production. An increased potential for urea degradation was suggested to promote gut microbiota dysbiosis and to exacerbate colitis in mice^33^. Conversely, allergy gut microbiome showed lower potential for complex fiber degradation, explaining the lower concentration of the SCFAs butyrate and propionate found in allergic subjects compared with healthy controls, as also observed in previous reports^14,15^. Therefore, the metagenome of allergic diseases is defined by an overall higher pro-inflammatory potential compared with healthy children, with an increased production of pro-inflammatory molecules, and a decreased biosynthesis of anti-inflammatory and tolerogenic SCFAs.

We identified specific signatures at sub-species levels linked with the allergic disease, suggesting the presence of a strain-level adaptation to the pro-inflammatory environment typical of the allergic condition. Indeed, we recognized a strain diversity linked to allergy in *Bif. longum* and *R. gnavus*. In particular, *R. gnavus* strains associated with allergy showed an enriched ability to adhere to the gut epithelium and colonize the gut environment, that may contribute to a pathogenic mechanism^34,35^, as well as a depletion of genes involved in complex polysaccharides break-down, contributing to the reduced concentration of SCFAs found in allergic children. These results support a previous hypothesis of a determinant role of *R. gnavus* in the development of allergy in the pediatric age^26^, but firstly highlighted that this association may be strain-dependent. Indeed, recent findings suggest that high variability at strain level exists in the gut microbiome and that different strains may be differently linked with health or diseases^20,36–38^. *R. gnavus* abundance increased with the consumption of an unhealthy diet rich in fat and animal products^39,40^. In addition, it was previously associated with inflammatory bowel diseases (IBD)^41,42^ and a recent study proposed a mechanism mediated by the production of an inflammatory polysaccharide, that was characterized as a glucorhamnan with a linear backbone formed from three rhamnose units and a short sidechain composed of two glucose units^22^. Accordingly, we identified a higher number of genes involved in the production of this polysaccharide^22^ in the gut metagenome of allergic children. In addition, Hall et al^42^. identified disease-specific clades of *R. gnavus* associated with IBD, characterized by higher adhesion potential to the gut epithelium, in line with our results. Consistently, we demonstrated that the fecal supernatant of allergic children was able to elicit the production of the Th2 cytokines IL-5 and IL-13 by human CD4+ T cells, although we cannot exclude that this response was due to the presence or co-presence of different compounds eliciting a pro-inflammatory effect.

Finally, we identified specific microbial signatures that may be involved in the resolution of FA after 36 months of exclusion diet. In a previous study, FA resolution at 8 years was linked with increased baseline abundance of Clostridia^17^. Thanks to a higher resolution, we identified higher abundance of *L. pectinoschiza* and *A. hadrus* (both Clostridia class), as well as of *Bif. longum*. Indeed, gut microbiome composition could predict the development of immune tolerance in a Random Forest classification model. This may suggest a possible implication of the gut microbiome in the immune tolerance acquisition pathways.

Using high-resolution metagenomics, we highlighted gut microbiome signatures (dysbiosis) in allergic children and strain-level adaptation in allergy, with *R. gnavus* emerging as likely involved in the pathogenesis of allergic disease. We also suggest that the production of pro-inflammatory molecules and the reduced ability to catabolize complex polysaccharides may be associated with the increased inflammation typical of allergic conditions. These findings support the importance of the gut microbiome in the onset of allergic diseases and may open new cues in the development of innovative preventive and therapeutic strategies based on microbiome manipulation.

## Methods

### Study subjects

Children (age range 48–84 months) with a sure diagnosis of IgE-mediated FA or RA, visiting our tertiary Center for Pediatric Allergy (www.allergologiapediatrica.eu), were considered for the study.

The exclusion criteria were: age at enrollment <48 or >84 months; history of non IgE-mediated allergy; eosinophilic disorders of the gastrointestinal tract; chronic systemic diseases; congenital cardiac defects; acute or chronic infections; autoimmune diseases; immunodeficiencies; chronic inflammatory bowel diseases; celiac disease; cystic fibrosis or other forms of primary pancreatic insufficiency; genetic and metabolic diseases; food intolerances; malignancy; chronic pulmonary diseases; malformations of the respiratory tract or of the gastrointestinal tract; pre-, pro- or sinbiotic use in the previous 3 months; antibiotics or gastric acidity inhibitors use in the previous 3 months. Written informed consent was obtained from the parents/caregiver of each child.

During the same study period, consecutive age-matched healthy children, with negative history for any allergic condition and not at risk for allergy, visiting our Department because of minimal surgical procedures or vaccination program were also enrolled. The same exclusion criteria were adopted.

Anamnestic, demographic, anthropometric and clinical data from each subject were recorded in a dedicated database. Subject recruitment and follow up were carried out at the Department of Translational Medical Science of the University Federico II, Naples, Italy. We collected from each study subjects two stool samples (3 g/each) on the same day before any therapeutic intervention for the allergic diseases. All stool samples were immediately stored at −80°C until analyses according to the Standard Operating Procedures (SOP 04) of the International Human Microbiome Standard Consortium.

All allergic patients were followed at the Center for at least 36 months after the enrollment. In children with FA, the possible acquisition of immune tolerance was assessed yearly by the results of skin prick tests, serum specific IgE levels and oral food challenge performed as previously described^43^. Similarly, healthy controls were followed by the physicians involved in the study for the possible occurrence of any allergic conditions for 36 months.

### Metagenome sequencing

DNA extraction from fecal samples was carried out following the SOP 07 developed by the International Human Microbiome Standard Consortium (www.microbiome-standards.org). DNA libraries were sequenced on Illumina NovaSeq platform, leading to 2x150bp, paired-end reads. Six stool samples failed in sequencing procedures, then shotgun metagenomics analysis was performed on 114 subjects: 30 with respiratory allergies (RA) (15 with allergic asthma and 15 with oculorhinitis), 55 with FA, ad 29 CT.

### Metagenomic reads filtering, taxonomic and functional analyses

Human reads were removed using the Human Sequence Removal pipeline developed within the Human Microbiome Project by using the Best Match Tagger (BMtagger; https://hmpdacc.org/hmp/doc/HumanSequenceRemoval_SOP.pdf). Then, non-human reads were quality-filtered using PRINSEQ 0.20.4:^44^ reads with bases having a Phred score < 15 were trimmed and those < 75 bp were discarded. Number of reads for each sample is reported in Supplementary Data 1. High-quality reads were imported in MetaPhlAn 3.0^45^ to obtain species-level, quantitative taxonomic profiles. Functional profiling was obtained using HUMAnN 3.0^46^. *Faecalibacterium* clade diversity was evaluated as recently described^20^, mapping short reads against a database of clade-specific marker genes^20^.

### Assembly free strain level analysis

PanPhlAn 3.0^45^ was applied on high-quality reads using default parameters, generating a presence/absence gene-family profiles for the top 11 most abundant species. Jaccard distance between each couple of samples was computed using *dist.binary* function (ade4 R package) and Classical Multidimensional Scaling (MDS, *cmdscale* function, stats R package) was carried out on Jaccard distance matrix.

### Assembly and genome reconstruction from metagenomics reads

High-quality reads were assembled independently using MEGAHIT v. 1.2.2^47^ and contigs >1000 bp were used to predict genes by using MetaGeneMark v. 3.26^48^. Assembly results are reported in Supplementary Data 1. Predicted genes were aligned (using BlastX – v. 2.2.30;^49^) against *Ruminococcus gnavus* genes coding for an inflammatory polysaccharide (as reported by^22^). An e-value cutoff of 1e^−5^ was applied, and a hit was required to display >95% of identity over at least 50% of the query length. In addition, we specifically focused on Carbohydrates-Active genes, aligning predicted genes against the CAZy database^50^ (non-redundant at 90% identity) by using DIAMOND v. 2.0.4^51^. An e-value cutoff of 1e^−5^ was applied, and a hit was required to display >90% of identity over at least 75% of the query length to be kept. The number of hits was normalized dividing by the total number of predicted genes in each sample.

Contigs (>1000 bp) were also binned using MetaBAT2 v. 2.12.1^52^, and Metagenome Assembled Genomes (MAG) quality was estimated with CheckM v. 1.1.3^53^. Only MAGs with >50% completeness and <5% contamination were retained for further analyses. MAGs binned in this study were clustered to a genomic database including 107,442 high-quality MAGs previously reconstructed from human metagenomes^54^ and 185,939 genomes from isolates downloaded from NCBI RefSeq on May 2020. Pairwise genetic distances between genomes were calculated using Mash (version 2.0; option “-s 10000” for sketching;^55^). A Mash distance <5% from any of the database genomes was considered to place the MAG within the relative Species-level Genome Bin (SGB). RAxML 8.0^56^ was used to generate species-specific phylogenetic trees, which were visualized in iTOL v. 5.5.1^57^.

### Fecal SCFAs determination

One gram of feces was diluted with saline buffer, vortexed and centrifuged (12,000 × *g*) for 10 min in 2 ml tubes. The supernatant was filtered (0.45 μm) and stored at −80°C until analysis. Frozen fecal extracts from −80°C were defrosted at 4°C for 12 h, then invert 10 times to mix at RT. One milliliter of each sample was acidified with 40 μl of H_3_PO_4_ 85% (w/v), vortexed for 5 min and sonicated for 10 min at 40 KHz immersed in an ice bath (Branson 2800 ultrasonic). One mL of ethyl acetate was added, vortexed for 10 min and centrifuged (12,000 RPM for 45 min). Finally, it was taken from the supernatant (organic phase, of about 1 mL) with a Pasteur pipette and placed in a new glass tube for gas-chromatography mass spectrometry (GC-MS) analysis. The GC column was an Agilent 122-7032ui (DB-WAX-U, Agilent Technologies, Santa Clara, California, USA) of 30 m, internal diameter of 0.25 mm, and film thickness of 0.25 μm. The GC was programmed to achieve the following run parameters: initial temperature of 50°C, hold of 1 min, ramp of 10°C min^−1^ up to a final temperature of 180°C, total run time of 20 min, gas flow of 70 ml min^−1^ splitless to maintain 12.67 p.s.i. column head pressure, and septum purge of 2.0 ml min^−1^. Helium was the carrier gas (1.5 ml min^−1^ costant). Parameters of mass spectrometer were: source at 230°C and MS Quad at 150 °C. The GraphPad PRISM 5 program was used to determine the concentration in mM. The data were inserted in the “XY” form in which in the “X” frame the values of the straight concentration-response were reported, while in the “Y” box the values of the area under the curve (AUC) related to the peaks obtained from the mass gas were reported. The AUC values of the single samples (obtained from the mass gas) were interpolated with the line X (concentration-response) to determine the corresponding mM concentration.

### Preparation of fecal supernatants

Fecal supernatants were obtained from stool samples of CT (*n* = 3, 2 male and 1 female, median age 52 months), FA (*n* = 3, 2 male and 1 female, median age 48 months), and RA (*n* = 3, 2 male and 1 female, median age 55 months) subjects, randomly selected from the dataset of our study population. Samples were prepared as previously described^58^. Briefly, phosphate buffered saline was added at equal volume to weight (1 g to 1 mL) and was homogenized into a suspension, which underwent three serial centrifugations at 1700, 19,000 and 35,200 × *g* for 15 min each using a Sorvall Rotor. The fecal supernatants were filtered with 0.22 µm filter for cell culture and stored at −80°C until use.

### Blood sampling and isolation of peripheral CD4+ T lymphocytes

Peripheral blood samples (8 ml) were obtained from three otherwise healthy children (Caucasian male, age range 48–61 months with negative clinical history for any allergic conditions and not at risk for atopic disorders), referred to the Department of Translational Medical Science at the University of Naples “Federico II” because of minimal surgical procedures. Peripheral mononuclear blood cells (PBMCs) were isolated by Ficoll density gradient centrifugation (Ficoll-Histopaque −1077, Sigma, St. Louis, Missouri, USA). Briefly, cells were stratified on 3 mL of Ficoll and centrifuged 15 min at 1000 × *g* at room temperature. After centrifugation, the opaque interface containing mononuclear cells was carefully aspirated with a Pasteur pipette and cells were washed with 10 mL of PBS and centrifuged 10 min at 500 × *g* at room temperature. After centrifugation, the upper layer was discarded and PBMCs were collected.

Naïve CD4+ T-cells were obtained by negative selection using the CD4+ T-Cell Isolation Kit II (Miltenyi Biotec, Bergisch Gladbach, Germany) from PBMCs. Non-target cells were labeled with a cocktail of biotin-conjugated monoclonal antibodies (MicroBead Cocktail, Miltenyi Biotec) and the magnetically labeled non-target T cells were retained on a column in the magnetic field of a separator (Miltenyi Biotec). This protocol produces >95% pure CD4+ T cells. Cells were cultured in duplicates in 96-well plates in 200 µL culture medium (RPMI 1640, Gibco) containing 10% FBS (Gibco), 1% non-essential amino acids (Gibco), 1% sodium pyruvate (Gibco), and 1% penicillin/streptomycin (Gibco).

### CD4+T cells stimulation protocol and Th2 cytokines determination

CD4+ cells (2 × 10^5^ cells/well) were stimulated with 5, 50, 100 µL of fecal supenatant for 1, 6, 18, and 24 h in time-course and dose-response experiments. Cells with only medium were used as negative control. After incubation period, culture supernatants were collected to assess the Th2 (interleukins IL-5 and IL-13) cytokines production. Concentrations of IL-5 and IL-13 in fecal supernatants and in CD4+ T cells culture media after stimulation, were measured using the IL-5 Human and IL-13 human ELISA kit from Elabscience (Elabscience, Houston, Texas). The detection limits were both 15.6 pg/ml.

### Statistical analyses

The Kolmogorov–Smirnov test was used to determine whether variables were normally distributed. Descriptive statistics were reported as the means and standard deviations for continuous variables, and discrete variables were reported as the number and proportion of subjects with the characteristic of interest. The χ2 test and Fisher’s exact test were used for categorical variables. To evaluate the differences among continuous variables, the two-tailed Student’s t test *t*-test were performed. The level of significance for all statistical tests was 2-sided, *p* < 0.05. All data were collected in a dedicated database and analyzed by a statistician using SPSS for Windows (SPSS Inc, version 23.0, Chicago, IL).

Differences in the overall gut microbiome taxonomic composition according to the disease status (CT vs RA + FA or CT vs FA or RA) were assessed by PERMANOVA (Permutational Multivariate Analysis of Variance, *adonis* function, vegan R package) computed on Jaccard distance matrix (*p <* 0.05). Comparisons of taxa or gene abundance between groups were carried out using pairwise Wilcoxon tests. Statistical significance of pangene prevalence was verified through Fisher’s test with multiple-hypothesis testing corrections via the false discovery rate (FDR).

Machine-learning-based classification analysis was done using the MetAML package^59^ and by considering random forests (RFs) as back-end classifier for all the experiments. Results were obtained through a five-fold cross-validation and averaged on 20 independent runs.

### Ethics approval and consent to participate

The study was conducted in accordance with the Helsinki Declaration (Fortaleza revision, 2013), the Good Clinical Practice Standards (CPMP/ICH/135/95), the Italian Decree-Law 196/2003 regarding personal data, and the European regulations on this subject. The study protocol, the subject information sheet and the informed consent form were reviewed and approved by the Ethics Committee of the University of Naples Federico II (approval N. 2/14). The study was registered in the Clinical Trials Protocol Registration System at ClinicalTrials.gov with the identifier NCT04750980.

### Reporting summary

Further information on research design is available in the Nature Research Reporting Summary linked to this article.

## Supplementary information

Supplementary information

Description of Additional Supplementary Files

Supplementary Data 1

Supplementary Data 2

Supplementary Data 3

Reporting Summary

## Data Availability

The raw sequence reads generated in this study have been deposited in the Sequence Read Archive (SRA) of the NCBI under accession number PRJNA706116. All softwares used for analyses are publicly available for download. CAzy database used in this study can be accessed from http://www.cazy.org; NCBI RefSeq genomes used in this study can be downloaded from https://ftp.ncbi.nlm.nih.gov/refseq/release/bacteria; human MAGs previously reconstructed^54^ and used in this study can be downloaded from http://segatalab.cibio.unitn.it/data/Pasolli_et_al.html. Source data are provided with this paper.

## Source data

Source Data
