# Supplementary material for: Specific gut microbiome signatures and the associated pro-inflamatory functions are linked to pediatric allergy and acquisition of immune tolerance
Source: Nat Commun. 2021 Oct 13;12:5958. doi: 10.1038/s41467-021-26266-z (PMC8514477; doi:10.1038/s41467-021-26266-z)
Supplement: Supplementary file 1 — Supplementary information [file 41467_2021_26266_MOESM1_ESM.pdf]

Specific gut microbiome signatures and the associated pro-inflammatory functions are linked to pediatric allergy and acquisition of immune tolerance

De Filippis F. et al.

Supplementary Information

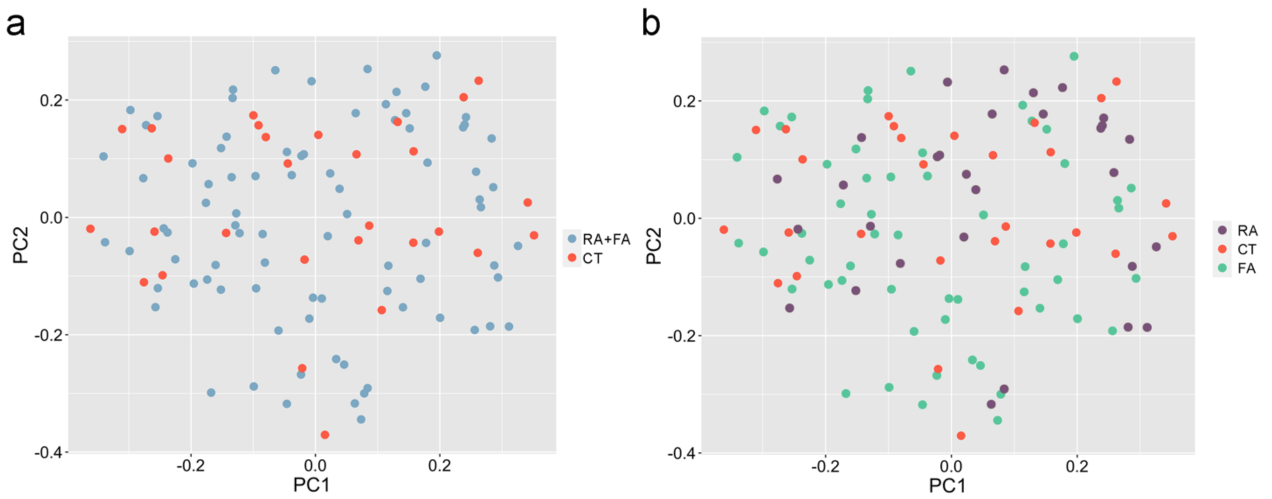

Figure S1. Overall gut microbiome composition does not discriminate allergic and healthy children.

Principal coordinates analysis based on the gut microbiome composition (as obtained by MetaPhlAn3). Allergic children are included in a unique group (a) or separated according to the type of allergy (b). CT, healthy controls; FA, food allergy; RA, respiratory allergy.

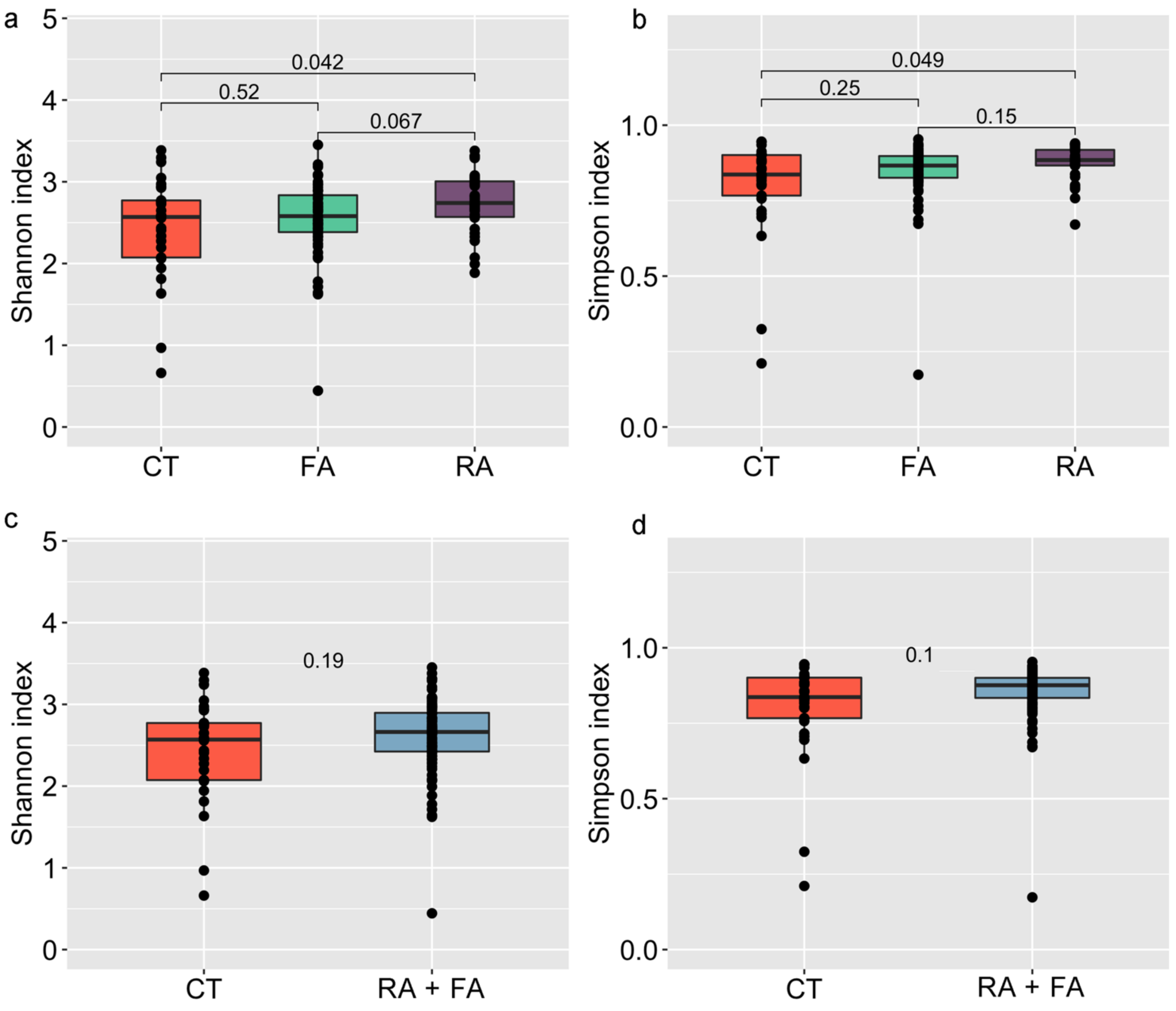

Figure S2. Respiratory allergy leads to higher gut microbial diversity.

Box plots showing Shannon (a, c) and Simpson (b, d) diversity index in allergic and healthy children. Allergic children are included in a unique group (c, d) or separated according to the type of allergy (a, b). The significance was tested by applying pair-wise Wilcoxon test. CT, healthy controls; FA, food allergy; RA, respiratory allergy. Boxes represent the interquartile range (IQR) between the first and third quartiles, and the line inside represents the median (2nd quartile). Whiskers denote the lowest and the highest values within 1.5 x IQR from the first and third quartiles, respectively. Data are obtained from n=29, 55 and 30 biologically independent samples for CT, FA and RA, respectively.

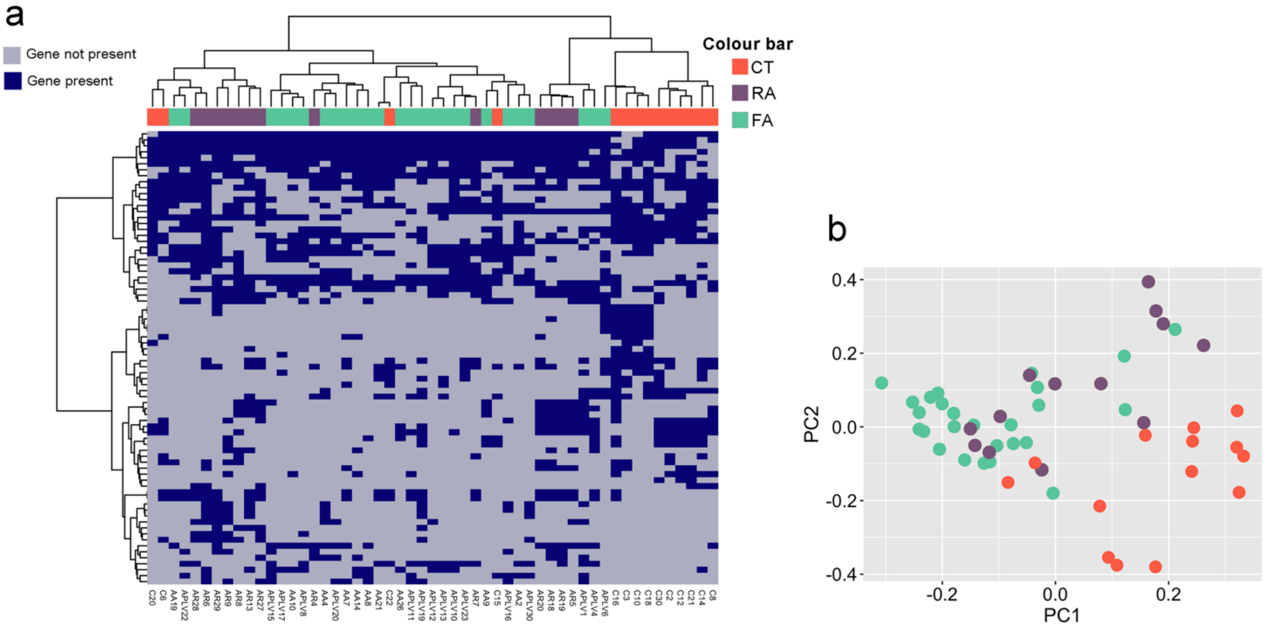

Figure S3. Allergic children harbor a different *B. bifidum* pangenome

a: Presence and absence of 76 *B. bifidum* genes significantly different between healthy (CT), food (FA) and respiratory (RA) allergic children (blue, present; gray, absent). The significance was tested by applying paired chi-squared tests; b: Principal coordinates analysis based on presence/absence of the 76 significant genes. The complete list of the 76 significant genes and their prevalence is reported in Supplementary Table S2B.

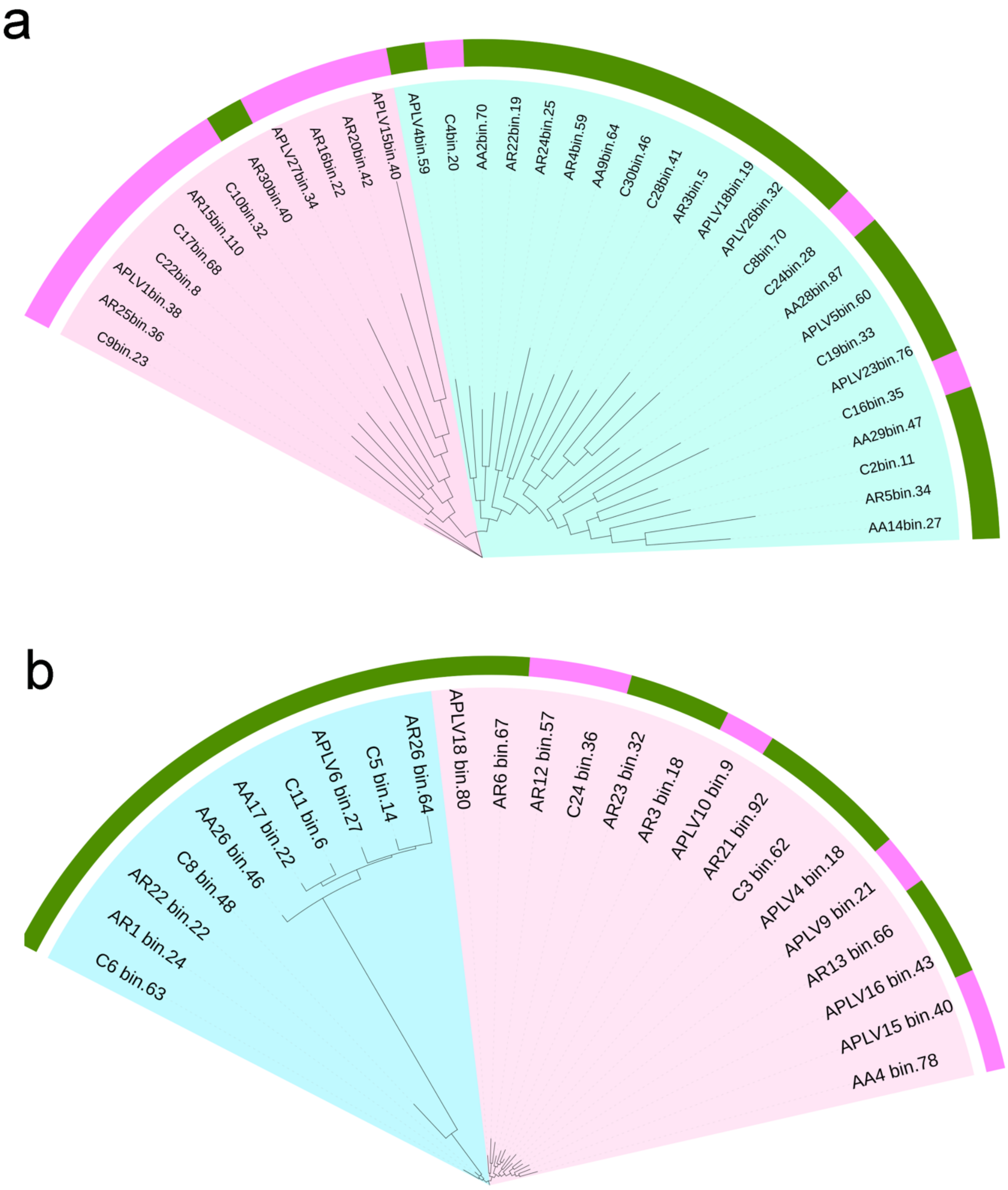

Figure S4. Delivery mode selects different *Blautia wexlerae* and *Bacteroides vulgatus* strains, independently from the health status.

Phylogenetic tree of the *Blautia wexlerae* (a) and *Bacteroides vulgatus* (b) Metagenome-Assembled Genomes (MAGs) retrieved from the children analysed. Outer ring is colored according to the delivery mode (pink, C-section; green, vaginal delivery).
