# Supplementary material for: Specific gut microbiome signatures and the associated pro-inflamatory functions are linked to pediatric allergy and acquisition of immune tolerance
Source: Nat Commun. 2021 Oct 13;12:5958. doi: 10.1038/s41467-021-26266-z (PMC8514477; doi:10.1038/s41467-021-26266-z)
Supplement: Supplementary file 2 — Description of Additional Supplementary Files [file 41467_2021_26266_MOESM2_ESM.docx]

**Specific gut microbiome signatures and the associated pro-inflammatory functions are linked to pediatric allergy and acquisition of immune tolerance**

**De Filippis F. et al.**

**Supplementary Data**

**Supplementary Data 1.** Sequencing and assembly statistics for the gut metagenomes.

**Supplementary Data 2.** Prevalence (%) of *B. bifidum* (A) and *R. gnavus* (B) pangenes significantly enriched or depleted (as defined by Fisher's test) in one group.

**Supplementary Data 3.** Taxonomic identification obtained for MAGs extracted from gut metagenomes.
