# Supplementary material for: Specific gut microbiome signatures and the associated pro-inflamatory functions are linked to pediatric allergy and acquisition of immune tolerance
Source: Nat Commun. 2021 Oct 13;12:5958. doi: 10.1038/s41467-021-26266-z (PMC8514477; doi:10.1038/s41467-021-26266-z)
Supplement: Supplementary file 6 — Reporting Summary [file 41467_2021_26266_MOESM6_ESM.pdf]

nature portfolio

Corresponding author(s): Danilo Ercolini, Roberto Berni Canani

Last updated by author(s): Sep 16, 2021

Reporting Summary

Nature Portfolio wishes to improve the reproducibility of the work that we publish. This form provides structure for consistency and transparency in reporting. For further information on Nature Portfolio policies, see our Editorial Policies and the Editorial Policy Checklist.

Statistics

For all statistical analyses, confirm that the following items are present in the figure legend, table legend, main text, or Methods section.

n/a	Confirmed	
☐	☒	The exact sample size (*n*) for each experimental group/condition, given as a discrete number and unit of measurement
☐	☒	A statement on whether measurements were taken from distinct samples or whether the same sample was measured repeatedly
☐	☒	The statistical test(s) used AND whether they are one- or two-sided<br>*Only common tests should be described solely by name; describe more complex techniques in the Methods section.*
☐	☒	A description of all covariates tested
☐	☒	A description of any assumptions or corrections, such as tests of normality and adjustment for multiple comparisons
☐	☒	A full description of the statistical parameters including central tendency (e.g. means) or other basic estimates (e.g. regression coefficient) AND variation (e.g. standard deviation) or associated estimates of uncertainty (e.g. confidence intervals)
☐	☒	For null hypothesis testing, the test statistic (e.g. *F*, *t*, *r*) with confidence intervals, effect sizes, degrees of freedom and *P* value noted<br>*Give P values as exact values whenever suitable.*
☒	☐	For Bayesian analysis, information on the choice of priors and Markov chain Monte Carlo settings
☒	☐	For hierarchical and complex designs, identification of the appropriate level for tests and full reporting of outcomes
☒	☐	Estimates of effect sizes (e.g. Cohen's *d*, Pearson's *r*), indicating how they were calculated

Our web collection on statistics for biologists contains articles on many of the points above.

Software and code

Policy information about availability of computer code

Data collection	NO SOFTWARE USED
Data analysis	Human Sequence Removal pipeline developed within the Human Microbiome Project by using the Best Match Tagger (BMtagger; https://hmpdacc.org/hmp/doc/HumanSequenceRemoval_SOP.pdf); PRINSEQ 0.20.4; MetaPhlAn 3.0; HUMAnN 3.0; PanPhlAn 3.0; MEGAHIT v. 1.2.2; MetaGeneMark v. 3.26; BlastX – v. 2.2.30; DIAMOND v. 2.0.4; MetaBAT2 v. 2.12.1; CheckM v. 1.1.3; RAxML 8.0 ; iTOL v. 5.5.1; R Studio 4.11

For manuscripts utilizing custom algorithms or software that are central to the research but not yet described in published literature, software must be made available to editors and reviewers. We strongly encourage code deposition in a community repository (e.g. GitHub). See the Nature Portfolio guidelines for submitting code & software for further information.

Data

Policy information about availability of data

All manuscripts must include a data availability statement. This statement should provide the following information, where applicable:

- Accession codes, unique identifiers, or web links for publicly available datasets
- A description of any restrictions on data availability
- For clinical datasets or third party data, please ensure that the statement adheres to our policy

Raw sequence reads produced in this study are available in the Sequence Read Archive (SRA) of the NCBI under accession number PRJNA706116. All softwares used for analyses are publicly available for download. CAzy database can be accessed from http://www.cazy.org; NCBI RefSeq genomes can be downloaded from https://ftp.ncbi.nlm.nih.gov/refseq/release/bacteria; human MAGs from [54] can be downloaded from http://segatalab.cibio.unitn.it/data/Pasolli_et_al.html.

Field-specific reporting

Please select the one below that is the best fit for your research. If you are not sure, read the appropriate sections before making your selection.

[x] Life sciences　[ ] Behavioural & social sciences　[ ] Ecological, evolutionary & environmental sciences

For a reference copy of the document with all sections, see nature.com/documents/nr-reporting-summary-flat.pdf

Life sciences study design

All studies must disclose on these points even when the disclosure is negative.

Sample size	We recruited all subjects visiting our centre in the considered period (January 1st 2017 to June 30th 2020) and meeting the inclusion criteria.
Data exclusions	One-hundred twenty children participated to the study (90 allergic children and 30 healthy controls). Six stool samples failed in sequencing procedures, then shotgun metagenomics analysis was performed on 114 subjects: 30 with respiratory allergies (RA) (15 with allergic asthma and 15 with oculorhinitis), 55 with FA, ad 29 CT.
Replication	Replicates reported in the text have to be considered as biological replicates, that is different subjects in the same study group. All analyses were carried out once.
Randomization	NOT APPLICABLE SINCE IT IS NOT A CLINICAL TRIAL
Blinding	NOT APPLICABLE SINCE IT IS NOT A CLINICAL TRIAL

Reporting for specific materials, systems and methods

We require information from authors about some types of materials, experimental systems and methods used in many studies. Here, indicate whether each material, system or method listed is relevant to your study. If you are not sure if a list item applies to your research, read the appropriate section before selecting a response.

Materials & experimental systems

n/a	Involved in the study	
[x]	[ ]	Antibodies
[x]	[ ]	Eukaryotic cell lines
[x]	[ ]	Palaeontology and archaeology
[x]	[ ]	Animals and other organisms
[ ]	[x]	Human research participants
[ ]	[x]	Clinical data
[x]	[ ]	Dual use research of concern

Methods

n/a	Involved in the study	
[x]	[ ]	ChIP-seq
[x]	[ ]	Flow cytometry
[x]	[ ]	MRI-based neuroimaging

Human research participants

Policy information about studies involving human research participants

Population characteristics	Children (age range 48-84 months) with a sure diagnosis of IgE-mediated FA or RA, visiting our tertiary Center for Pediatric Allergy (www.allergologiapediatrica.eu), were considered for the study.<br>The exclusion criteria were: age at enrollment <48 or >84 months; history of non IgE-mediated allergy; eosinophilic disorders of the gastrointestinal tract; chronic systemic diseases; congenital cardiac defects; acute or chronic infections; autoimmune diseases; immunodeficiencies; chronic inflammatory bowel diseases; celiac disease; cystic fibrosis or other forms of primary pancreatic insufficiency; genetic and metabolic diseases; food intolerances; malignancy; chronic pulmonary diseases; malformations of the respiratory tract or of the gastrointestinal tract; pre-, pro- or sinbiotic use in the previous 3 months; antibiotics or gastric acidity inhibitors use in the previous 3 months. Written informed consent was obtained from the parents/caregiver of each child. About 70% and 62% of the subjects were male, in RA and FA groups, respectively.<br>During the same study period, consecutive age-matched healthy children, with negative history for any allergic condition and not at risk for allergy, visiting our Department because of minimal surgical procedures or vaccination program were also enrolled. The same exclusion criteria were adopted. Males were the 52% of the control subjects.<br>Baseline main demographic and clinical characteristics of the study population were reported in Table 1.
Recruitment	We didn't find any potential self-selection bias or other biases that may had an impact on study results. We evaluated for the study all children (age range 48-84 months) with IgE-mediated FA or RA, consecutively observed at our tertiary Center for Pediatric Allergy (www.allergologiapediatrica.eu). Only patients with a sure diagnosis of FA or RA were included in the study. In all subjects the diagnosis was obtained according to validated criteria.
Ethics oversight	The study was conducted in accordance with the Helsinki Declaration (Fortaleza revision, 2013), the Good Clinical Practice Standards (CPMP/ICH/135/95), the Italian Decree-Law 196/2003 regarding personal data, and the European regulations on this subject. The study protocol, the subject information sheet and the informed consent form were reviewed and approved by the Ethics Committee of the University of Naples Federico II (approval N. 2/14).

Note that full information on the approval of the study protocol must also be provided in the manuscript.

Clinical data

Policy information about clinical studies

All manuscripts should comply with the ICMJEguidelines for publication of clinical research and a completedCONSORT checklist must be included with all submissions.

Clinical trial registration	The study was registered in the Clinical Trials Protocol Registration System at ClinicalTrials.gov with the identifier NCT04750980.
Study protocol	ClinicalTrials.gov with the identifier NCT04750980
Data collection	Enrollement and clinical data collection were carried out from June 1st 2017 to June 30th 2020, at the Center for Pediatric Allergy (www.allergologiapediatrica.eu) of the University Hospital Federico II, Naples (Italy)
Outcomes	PRIMARY OUTCOME: Evaluation of gut microbiome features in allergic children and in healthy controls.
